# Supplementary figures and images for: Arginase 2 Deficiency Promotes Neuroinflammation and Pain Behaviors Following Nerve Injury in Mice
Source: J Clin Med. 2020 Jan 22;9(2):305. doi: 10.3390/jcm9020305 (PMC7073606; doi:10.3390/jcm9020305)

Supplementary Figure 1 Print area

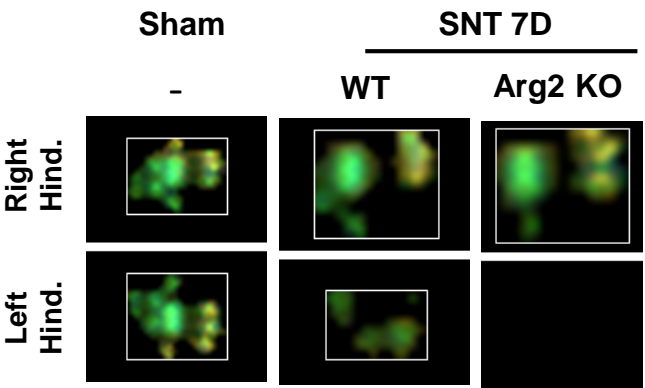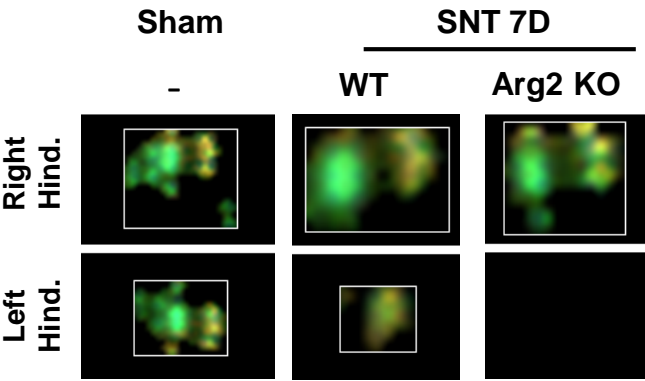

Supplementary Figure 2    Single stance

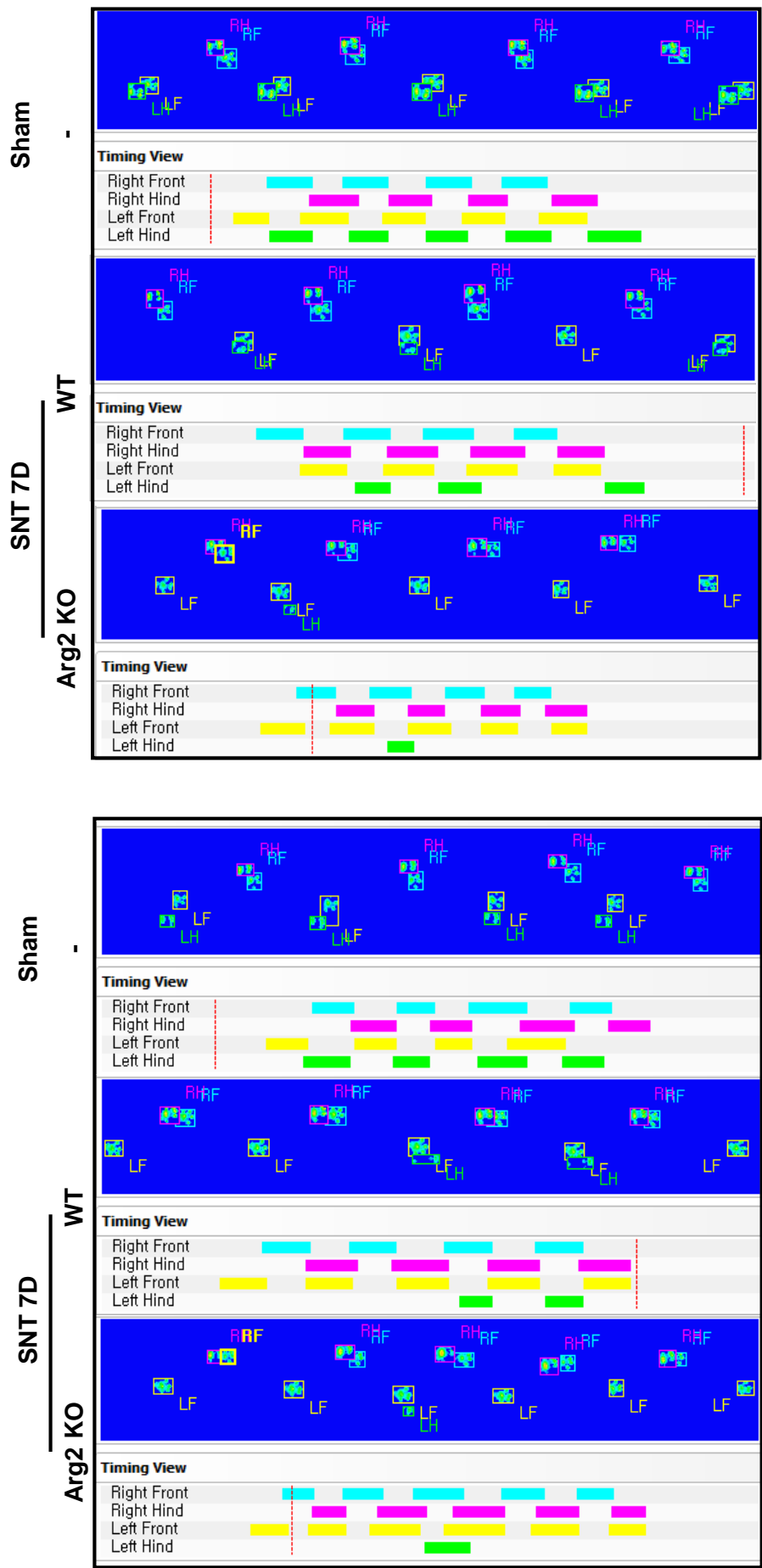

Supplement: Supplementary file 1 [file jcm-09-00305-s001.pdf]
